# Editorial: Combating Diabetes and Diabetic Kidney Disease

**DOI:** 10.3389/fphar.2021.716029

**Published:** 2021-07-08

**Authors:** Swayam Prakash Srivastava, Keizo Kanasaki, Julie E. Goodwin

**Affiliations:** ^1^ Department of Pediatrics, Yale University School of Medicine, New Haven, CT, United States; ^2^ Vascular Biology and Therapeutics Program, Yale University School of Medicine, New Haven, CT, United States; ^3^ Internal Medicine 1, Shimane University Faculty of Medicine, Izumo, Japan

**Keywords:** diabetes, diabetic kidney disease, microRNAs, SGLT-2 inhibitors, DPP-4 inhibitors, EndMT, long noncoding RNAs, mineralocorticoid receptor antagonism

Diabetic kidney disease (DKD) is a leading cause of end-stage renal disease, resulting in more than 950,000 deaths each year globally (Thomas et al., 2015; Cooper and Warren, 2019). These patients carry a significantly increased risk of cardiovascular morbidity and mortality. The link between renal disease and cardiovascular disease is poorly understood and this knowledge gap contributes to the suboptimal treatment options available for these patients. Improved understanding of the pathogenesis of DKD and its association with the development of cardiovascular disease is urgently needed to catalyze the development of novel therapeutics and should be targeted to the early stages of these diseases, before kidney and/or cardiovascular damage becomes irreversible.

Currently approved therapeutic regimens include ACE inhibitors, angiotensin receptor blockers (ARBs), and statins which minimize, but do not prevent, the progression of cardiovascular morbidities and the incidence of ESRD (Srivastava et al., 2020a; Hartman et al., 2020). Moreover, these therapies are neither tissue- nor cell-specific and are ineffective in reversing kidney fibrosis and diabetic complications. In recent years, a number of reno-protective agents, including sodium glucose co-transporter (SGLT-2) inhibitors, mineralocorticoid receptor antagonists, endothelin A antagonists, dipeptidyl transferse-4 (DPP-4) inhibitors, and N-seryl-acetyl-lysyl-proline have been studied in both preclinical settings and in controlled clinical trials, some with promising outcomes (Kanasaki et al., 2014; Stavropoulos et al., 2018; Srivastava et al., 2020b). Still, more research is needed to validate their cell- and tissue-specific mechanisms to optimize their use in human disease. Understanding these critical pathways will guide future therapies to combat kidney fibrosis and cardiovascular complications in diabetes.

In this special issue of Frontiers in Pharmacology, we discuss new pathophysiologic mechanisms which are driving therapies to combat kidney fibrosis in diabetes. We focused on three major sections.

## New Leads Targeted to DKD

First, we discuss new leads targeted to DKD. In recent years SGLT-2 inhibitors are of significant importance in restoring kidney structure and fibrotic phenotypes in diabetes. SGLT-2 plays a key role in reabsorption of glucose filtered from the glomerulus. Nearly all (90–95%) filtered glucose in the urine is reabsorbed through SGLT2. The EMPA-REG trial demonstrated that the SGLT2 inhibitor empagliflozin reduced renal complications in high-risk diabetic patients and was also effective in patients with advanced kidney disease; this finding represents a key development which advances the clinical practice of diabetic medicine (Mayer et al., 2019). These researchers explain that the renal benefit of SGLT2 inhibition is based on hemodynamic alterations and the ability to lower blood glucose. However, SGLT2 inhibitors might also protect the kidneys from defective central metabolism as evidenced by their ability to mitigate abnormal glycolysis and improve lipid metabolism (Li et al., 2020b). In this issue, a meta-analysis and randomized clinical trials demonstrate the beneficial effect of SGLT-2 inhibitors on hemoglobin and hematocrit levels, suggesting that SGLT-2 inhibitors treatment may offer additional benefit in DKD (Qu et al.). Similarly, DPP-4 inhibitors such as linagliptin, and incretin analogs, which are known drugs for treatment of type II diabetes, are effective in improving kidney fibrosis in diabetes in preclinical settings. Various DPP-4 inhibitors have diverse effects in kidney health and are dependent on specific drug types and metabolic characteristics. Research led by Professor Kawanami discusses the beneficial effects and clinical efficacy of glucagon-like-peptide-1 (GLP-1) agonists in DKD (Kawanami and Takashi.). GLP-1 agonists have the potential to develop into a future class of medication for combating DKD. Another review article describes new therapeutic targets such as DPP-4, notch signaling, and sirtuins in DKD (Zoja et al.).

## New Cellular Mechanisms in DKD

In recent years research by our group (Yale University, United States; Kanazawa Medical University, Japan) has focused on mesenchymal metabolic shifts that play a critical role in renal fibrosis (Li et al., 2020a; Srivastava et al., 2021a). Abrogation of both defective central metabolism and mesenchymal metabolic shifts through the use of small chemicals (glycolysis inhibitors and fatty acid oxidation activators) is effective in improving kidney structure and function (Kang et al., 2015; Srivastava et al., 2018). Glucocorticoid receptors (GR) are essential for endothelial cell homeostasis and regulate defective metabolism in endothelial cells. Endothelial GR regulates renal fibrogenesis by targeting Wnt signaling, defective fatty acid oxidation and associated mesenchymal activation in diabetic kidneys (Srivastava et al., 2021b). In this issue, the authors discuss new cellular mechanisms and cell signaling in the regulation of DKD pathogenesis. In this section, we describe the significance of mitochondrial control for the health and metabolism of the kidneys. Mitochondrial SIRT3 regulates cell-to-cell differentiation programs in kidney endothelial cells and its deficiency influences cellular *trans*-differentiation processes in neighboring cells, suggesting that SIRT3 is crucial for cellular homeostasis in diverse cell types in the kidney (Srivastava et al.). Sol et al., describe the importance of glomerular endothelial cells in sclerotic glomerular diseases such as focal segmental glomerulosclerosis and diabetic nephropathy (Sol et al.). Another article describes the differences in molecular and cellular mechanisms of ROCK1 and ROCK2 in DKD and discusses how targeting ROCK1 and ROCK2 have shown beneficial effects in treating other microvascular complications such as neuropathy and retinopathy (Matoba et al.). Sheng et al., describe the functional role of epidermal growth factor receptor (EGFR) in the development of DKD and discuss the therapeutic potential of EGFR inhibitors in the treatment of DKD (Sheng et al.). In brief, authors describe that the persistent activation of EGFR causes hemodynamic alterations, metabolic disturbances, inflammatory responses and parenchymal cellular dysfunction (Sheng et al.). Furthermore, an article describes the critical roles of FOXO1 in the regulation of cellular homeostasis and post-translational modifications (Wang et al.). The authors discuss how FOXO1 dysregulation contributes to the development of DKD and how improvement in FOXO1 dysregulation is associated with reversal of DKD phenotypes. Hence, FOXO1 is a potential therapeutic target in DKD (Wang et al.).

## Non-coding RNAs in DKD

MiR-29 and miR-let-7 family clusters are the key antifibrotic microRNAs which are regulated by cross-talk mechanisms in endothelial cells, and this cross-talk regulation protects against endothelial-to-mesenchymal transition (Srivastava et al., 2016). Also, crosstalk regulation inhibits pro-fibrotic mechanisms (i.e. DPP-4 level and TGFβ signaling) and regulates health and disease processes of diverse type of kidneys cells. miR-29 and miR-let-7 family clusters require further exploration in diabetic nephropathy (Srivastava et al., 2019). In this issue, research led by Shi et al., adds further useful information about interactions between long-noncoding RNAs and microRNAs in endothelial cells (Shi et al.). Such interactions are physiologically important in renal health and disease processes under diabetic conditions, in which expression of LncRNA-H19 is higher and concomitantly inhibits the anti-mesenchymal and protective effect of miR-29a, resulting in more fibrosis. Under non-diabetic conditions, miR-29a binds to LncRNA-H19 and inhibits its profibrotic properties, resulting in less fibrosis. Moreover, lncRNAs-H19 function as sponges for miR-29a to regulate the expression of its target proteins. Another review describes new therapeutic strategies and the role of anti-fibrotic and pro-fibrotic microRNAs in DKD (Sakuma et al.). The authors discuss the antifibrotic roles of miR-29 and miR-let-7s and the pro-fibrotic roles of miR-21 and miR-214 in multiple dimensions of DKD. Further, Gu et al., add the functional importance of non-coding RNAs and discuss their potential as biomarkers in DKD (Gu et al.). Further research is needed to translate their potential to the clinical setting.

## Conclusion

Diabetic kidney fibrosis is an important research topic for both clinicians and research scientists. In this special issue, we have discussed recent therapeutic advancements and new drug targets for combating kidney fibrosis and vasculopathy in diabetic nephropathy. We hope this special issue provides useful information for clinicians and basic science researchers to catalyze novel therapeutic approaches and future research directions.
